# Calcium Phosphate Honeycomb Scaffolds with Tailored Microporous Walls Using Phase Separation-Assisted Digital Light Processing

**DOI:** 10.3390/ma18112587

**Published:** 2025-06-01

**Authors:** Gyu-Nam Kim, Jae-Hyung Park, Jae-Uk Song, Young-Hag Koh, Jongee Park

**Affiliations:** 1 School of Biomedical Engineering, Korea University, Seoul 02841, Republic of Korea; gyunamkd@korea.ac.kr (G.-N.K.); jhbest210@korea.ac.kr (J.-H.P.); jx0582@korea.ac.kr (J.-U.S.); 2 Interdisciplinary Program in Precision Public Health, Korea University, Seoul 02841, Republic of Korea; 3 Department of Metallurgical and Materials Engineering, Atilim University, Ankara 06830, Türkiye; jongee.park@atilim.edu.tr

**Keywords:** digital light processing, camphene, phase separation, porous ceramic scaffolds, varying porosity

## Abstract

The present study reports on the manufacturing of biphasic calcium phosphate (BCP) honeycomb scaffolds with tailored microporous walls using phase separation-assisted digital light processing (PS-DLP). To create micropores in BCP walls, camphene was used as the pore-forming agent for preparing BCP suspensions, since it could be completely dissolved in photopolymerizable monomers composed of triethylene glycol dimethacrylate (TEGDMA) and polyethylene glycol diacrylate (PEGDA) and then undergo phase separation when placed at 5 °C. Therefore, solid camphene crystals could be formed in phase-separated BCP layers and then readily removed via sublimation after the photopolymerization of monomer networks embedding BCP particles by DLP. This approach allowed for tight control over the microporosity of BCP walls by adjusting the camphene content. As the camphene content increased from 40 to 60 vol%, the microporosity increased from ~38 to ~59 vol%. Consequently, the overall porosity of dual-scale porosity scaffolds increased from ~51 to ~67 vol%, while their compressive strength decreased from ~70.4 to ~13.7 MPa. The mass transport ability increased remarkably with an increase in microporosity.

## 1. Introduction

Calcium phosphate (CaP) ceramics have been extensively employed to produce bone grafts for repairing and regenerating damaged bones in dentistry and orthopedics, since they can mimic the chemical compositions and biological functions of the inorganic phase (Ca-deficient hydroxyapatite; HA) of natural bones [1,2,3,4,5,6,7]. More specifically, they can be highly biocompatible with neighboring bone tissues without any undesired foreign responses when implanted into bone defects, and new bone tissue formation is stimulated due to ion release (e.g., Ca^2+^, PO_4_^3−^, and OH^−^) when exposed to body fluids [1,3,4,5,6]. Furthermore, β-tricalcium phosphate (β-TCP) exhibits significantly higher resorbability and biodegradability compared to hydroxyapatite (HA), allowing for rapid ion release and subsequent replacement by natural bone tissue [3,4,5,6]. In contrast, HA degrades at a much slower rate, providing long-term structural support. Therefore, the combination of β-TCP and HA in biphasic calcium phosphate (BCP) ceramics offers a balance between bioactivity and mechanical integrity, making BCP particularly suitable for bone regeneration and implant applications. In addition, to accelerate bone regeneration, CaP-based bone grafts are generally formulated into porous structures with large pores (e.g., >100 μm in size), which are referred to as “bone scaffolds” [2,3,4,5,8,9,10,11,12,13,14]. More specifically, a number of bone-forming cells can migrate favorably through pores and adhere to spacious surfaces, followed by cell proliferation and differentiation for new bone tissue formation [10,12,15,16,17]. However, the mechanical properties of porous bone scaffolds decrease severely with an increase in porosity, which hinders the clinical usage of porous bone scaffolds as repairing load-bearing bones (e.g., vertebrae and long leg bones) that should withstand considerable compressive strengths and strains caused by gravitational force during daily life [18,19].

Therefore, considerable efforts have been made to overcome the trade-off between porosity and mechanical properties of porous ceramic bone scaffolds by controlling their porous structures [18,19,20,21,22]. In this respect, additive manufacturing (AM) techniques have gained special attention, since they can construct arbitrarily designed pore geometries in a highly controlled manner [8,9,10,11,12,13,14,15,23,24], which enhanced by a considerable amount the mechanical properties and reliability when comparing to traditionally used porous structures with randomized pore orientation. As the AM technique, material extrusion (ME) is useful to construct periodically aligned pores with tailored orientations (e.g., 0°/90° and 0°/60°/120°) by depositing filaments made of ceramic suspensions extruded through fine nozzles according to predetermined build paths, followed by the densification of ceramic filaments at high temperatures. In addition, more sophisticated porous structures with a high degree of design freedom can be achieved by employing vat photopolymerization (VP) techniques (e.g., digital light processing (DLP) and stereolithography (SLA)) [8,9,12,14,15,23,24]. They can selectively photopolymerize the thin layers of highly concentrated ceramic suspensions using high-resolution light modules (e.g., digital micromirror device (DMD) for DLP). A variety of porous structures can be constructed, including a cubic lattice [25,26,27], woodpile lattice [28,29,30], diamond lattice [15,31,32], truncated octahedral lattice [15,25,27], and triply periodic minimal surfaces (TPMSs) [15,33,34,35,36,37]. This great ability can provide opportunities for designing novel types of porous BCP bone scaffolds with considerably enhanced mechanical properties and bone regeneration abilities.

In addition, the use of ceramic suspensions containing pore-forming agents specially formulated for ME and VP techniques can create micron-scaled pores (herein denoted as “micropores”) within ceramic frameworks [24,28,29,33,38,39,40,41,42,43]. By using ceramic suspensions with pore-forming agents, dual-scaled porous ceramics composed of macropores surrounded by microporous frameworks can be manufactured, which mainly aim to provide significantly enhanced mechanical properties at given porosities compared to single-scaled porous structures [28,29,33,38,39,40,41,42]. In addition, micropores additionally formed within ceramic frameworks can facilitate the transport of body fluids, blood, growth factors, oxygen, and nutrients for cellular response, while macropores can effectively induce fast bone ingrowth [16,44,45,46,47,48]. As the pore-forming agent, polymeric particulates can be readily mixed with ceramic suspensions and then completely removed by thermal decomposition during the heat-treatment of green objects manufactured by AM [33,38,40,41,42,43]. However, this approach often results in narrow interconnection between micropores due to the limited contents of polymeric particulates. In other words, the viscosity of ceramic suspensions would increase with an increase in particulate content, which makes it impractical for VP processes. Unlike solid pore-forming agents, liquid droplets and air bubbles can be incorporated into ceramic suspensions specifically for achieving exceedingly high porosities [49,50,51,52]; nonetheless, the limited pore interconnectivity remains challenging.

On the other hand, several types of liquid media (e.g., water and camphene) used to prepare ceramic suspensions can serve as pore-forming agents [53,54,55,56,57,58]. More specifically, they can be converted into solid crystals when placed below their freezing points (0 °C for water and ~51 °C for camphene), while pushing ceramic particles to be concentrated between crystals. Consequently, three-dimensional pores can be created by removing solid crystals via sublimation during the freeze-drying of green ceramic objects manufactured by AM processes. In addition, liquid camphene, which is molten at elevated temperatures (e.g., ≥70 °C), can be uniformly blended with several types of photocurable monomers and ceramic particles to formulate ceramic suspensions, which can be used for photocuring-assisted ME [43,56,59,60] and VP processes [28,29]. More specifically, when placed at room temperature, monomers enclosing ceramic particles can be concentrated between camphene and then effectively photopolymerized by UV light. More recently, our group demonstrated that solid camphene can be dissolved in several types of photocurable monomers, including 1,6-hexanediol diacrylate (HDDA) [58,61] and triethylene glycol dimethacrylate (TEGDMA) [62,63] and then undergo unique phase separation at temperatures slightly lower than room temperature. This approach using camphene/photocurable monomer solutions as the phase-separable, photocurable vehicles for DLP process, herein denoted as “phase separation-assisted DLP (PS-DLP)”, can allow for designing a variety of dual-scale porosity ceramics with tailored macroporous and microporous structures.

**Figure 1:**
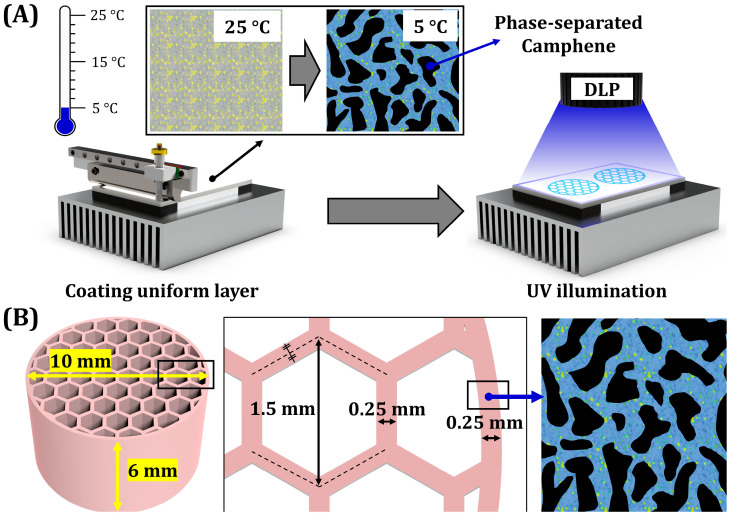
(**A**) Schematic diagram showing working principle of phase separation-assisted DLP process and (**B**) honeycomb structured scaffold composed of macro-sized channels with micro-sized pores.

This study demonstrates the utility of PS-DLP for manufacturing dual-scale porosity BCP ceramics particularly to be used as load-bearing bone scaffolds. A schematic diagram showing the overall PS-DLP process is shown in Figure 1A. To this end, a honeycomb structure composed of unidirectionally aligned macropores is employed, since it can provide high compressive strengths and stiffness due to aligned frameworks and outstanding bone regeneration abilities in vivo [50,64,65,66]. Moreover, our honeycomb scaffolds are designed to have three-dimensionally interconnected micropores within frameworks for accelerating new bone formation (Figure 1B). For the PS-DLP process, TEGDMA monomer was blended with polyethylene glycol diacrylate (PEGDA) based on our previous report [63]. To tailor the microporosity of the BCP frameworks, the camphene content in TEGDMA/PEGDA blends was adjusted in the range of 40–60 vol%, and the content of each TEGDMA and PEGDA monomer was controlled. By the PS-DLP process, after repeating the recoating and selective UV-light curing step, BCP scaffolds with honeycomb structure were prepared. Dual-scale porosity structures and microporous structures of BCP frameworks were carefully examined. In addition, the mechanical properties of dual-scale porosity BCP scaffolds were evaluated by compressive strength tests to examine their potential as load-bearing bone scaffolds. Therefore, the aim of this study is to fabricate BCP ceramic scaffolds with various dual-scale porosities using PS-DLP technique and to systematically investigate the effect of camphene content on their microstructure, mechanical strength, and transport properties.

## 2. Materials and Methods

### 2.1. Compositions of BCP Suspensions

The starting material for this study was a commercially available biphasic calcium phosphate (BCP) powder (Future Institute of Materials Science Co. Ltd., Suwon-si, Gyeonggi-do, Republic of Korea), consisting of 60 wt% hydroxyapatite (HA, Ca_10_(PO_4_)_6_(OH)_2_) and 40 wt% β-tricalcium phosphate (β-TCP, β-Ca_3_(PO_4_)_2_). Camphene (C_10_H_16_, Sigma-Aldrich, Saint Louis, MO, USA) served as the pore-forming agent. The photopolymerizable monomers used were triethylene glycol dimethacrylate (TEGDMA, Tokyo Chemical Industry Co., Ltd., Tokyo, Japan) as the solvent and poly(ethylene glycol) diacrylate with a molecular weight of ~575 (PEGDA, Sigma Aldrich, Saint Louis, MO, USA) as the anti-solvent. DISPERBYK-180 (BYK-Chemie Inc., Kempen, Germany) was used as the dispersant. Diphenyl(2,4,6-trimethylbenzoyl)phosphine oxide (TPO, Sigma Aldrich, Saint Louis, MO, USA) served as the photoinitiator. Finally, Oil Red O (Sigma Aldrich, Saint Louis, MO, USA) was incorporated as an inert dye to achieve high resolution.

### 2.2. Preparation of BCP Suspensions

Phase-separable BCP suspensions with varying camphene contents (40, 50, and 60 vol%) were prepared by dissolving camphene in a photopolymer blend of TEGDMA and PEGDA at different ratios. The weight fractions of the camphene and photopolymer blend for all suspensions are summarized in Table 1, following the compositional ratios based on a previous study [63]. Initially, camphene, TEGDMA, and PEGDA were combined to form homogeneous mixtures. Subsequently, BCP powder (33.8 vol% relative to the total suspension volume excluding camphene) was introduced into the camphene/TEGDMA-PEGDA solution along with the dispersant (5 wt% relative to the BCP content, based on the previous study [63]). This mixture underwent a paste-mixing process for 30 min at 1000 rpm using a planetary centrifugal mixer (Hantech Co, Ltd., Suwon-si, Gyeonggi-do, Republic of Korea). Prior to the DLP process, the photoinitiator (2 wt% relative to the total monomer content) and a small quantity of inert dye were added, followed by an additional 20 min of mixing at 1000 rpm.

### 2.3. Characterization of BCP Powder and Suspensions

The morphology and particle size distribution of BCP particles were examined using field emission scanning electron microscopy (FE-SEM; JSM-6701F, JEOL Techniques, Tokyo, Japan) and a laser diffraction particle size analyzer (LA-350, Horiba, Kyoto, Japan), respectively. The crystalline phases of the BCP powder were characterized by X-ray diffraction with measurement conditions of 3 °/min, 40 kV, and 40 mA (XRD; D8 Advance, Bruker, Billerica, MA, USA). Rietveld refinement was performed using XRD analysis software (Diffrac. Topas, Version 5.0, Bruker, Billerica, MA, USA) to perform quantitative analysis on the contents of HA and β-TCP phases coexisting in BCP particles. The residual was calculated by subtracting the calculated diffraction pattern from the experimental values.

The phase separation behavior of the three BCP suspensions with different camphene contents was evaluated using differential scanning calorimetry (DSC, TA Instruments Q20, TA Instruments, New Castle, DE, USA) under a nitrogen atmosphere. The suspensions were cooled from 30 °C to 0 °C at a cooling rate of 1 °C/min to observe the crystallization of camphene within the mixture. Heat flows during DSC were recorded as a function of temperature.

The rheological properties of the three BCP suspensions were assessed by measuring their apparent viscosities as a function of shear rate (0.1 s^−1^–200 s^−1^) using a cone/plate rheometer (DVNext, Brookfield Engineering Laboratories, Inc., Middleborough, MA, USA) at 25 °C.

To determine the optimal photo-curing time for the phase separation-assisted DLP 3D printing process, layers of BCP suspensions with different camphene contents were placed at a temperature of 5 °C (based on the DSC analysis of BCP suspensions) and then UV light was exposed with the DLP engine to induce photopolymerization for various exposure times. The platform was covered with black tape to prevent curing by reflected light. Subsequently, the thickness of the photopolymerized layers was measured using a micrometer.

The evaluations for XRD, DSC, rheology, and photo-curing time were conducted once each for BCP powder and all BCP suspensions with different camphene contents (*n* = 1).

### 2.4. Digital Light Processing (DLP) Fabrication

A custom-built DLP machine developed in our group was used for the fabrication of BCP scaffolds [63]. The machine was equipped with a digital micromirror device (PRO4710, Wintech Digital System Technology Corp., San Marcos, CA, USA) with a light intensity of 10.7 mW/cm^2^ at a peak wavelength of 405 nm. The resolution in the x-y plane was approximately 35.5 μm. The thermoelectric modules were attached to the build platform to cool and control the temperature at which the suspensions were recoated.

BCP suspensions were deposited onto a build platform maintained at 5 °C. Note that a relatively low temperature of 5 °C was employed based on DSC analyses to induce phase separation of the camphene within BCP suspensions and to avoid condensation of water vapor at the surface of the platform during PS-DLP process. After the deposition of BCP suspension, a uniform layer of suspension with a thickness of 100 μm was spread using a recoater. After recoating, each layer was exposed to UV light with a corresponding sliced 2D image for 6.5–8 s to induce photopolymerization. The power applied to the thermoelectric module gradually increased with the number of printed layers to maintain similar temperatures for phase separation of newly formed layers. This process was repeated to construct the designed honeycomb structure with a diameter of 10 mm and a height of 6 mm. The size of a unit cell was 1.5 mm with a framework width of 0.25 mm. Furthermore, the dimensions of as-manufactured samples, including diameters and heights, were measured for further investigation.

### 2.5. Post-Processing and Sintering

Following the printing process, the as-manufactured BCP scaffolds with different camphene contents were carefully detached from the platform and rinsed manually with ethanol. This was followed by ultra-sonification to thoroughly remove any uncured, residual suspensions. After rinsing, the samples underwent freeze-drying for 24 h to remove the camphene dendrites, resulting in a porous framework of BCP particles within a solid photopolymer matrix.

The samples underwent a careful multi-step debinding process in a furnace (Ajeon Heating Industrial Co., Ltd., Namyangju-si, Gyeonggi-do, Republic of Korea) to eliminate organic materials while minimizing any defects during the heating process. The debinding schedule included a heating rate of 1 °C/min up to 440 °C. After debinding, the samples were sintered at 1150 °C for 3 h with a heating rate of 5 °C/min to densify the BCP walls while preserving the macroporous and microporous structure. Note that such a high temperature of 1150 °C for the sintering process was employed to induce densification between BCP particles within the walls. The multi-step heating schedule is presented in Table 2.

### 2.6. Characterization of Scaffolds

The dimensions of the sintered scaffolds, including the diameter (D) and height (H), were measured before and after sintering at 1150 °C for 3 h to determine sintering shrinkage (*n* = 5 for each BCP specimen with different camphene contents). The percentage shrinkage in both the x-direction and z-direction was calculated using the following equation:

(1) $$Linear\;Shrinkage\;\left[\%\right]=100\;\cdot \left(\frac{Initial\;Dimension-Sintered\;Dimension}{Sintered\;Dimension}\right)$$

The crystalline phases of sintered BCP specimens were characterized using X-ray diffraction to observe the change in crystalline phases after sintering at 1150 °C for 3 h (*n* = 1 for each BCP specimen with different camphene contents). Observed peaks were then identified by considering diffraction patterns (diffraction angles and relative intensities) of the HA (JCPDS card no. 00-009-0432) and β-TCP (JCPDS card no. 04-014-2292) phases of BCP. Rietveld refinement was performed using XRD analysis software (Diffrac. Topas, Bruker, Billerica, MA, USA) to perform quantitative analysis on the contents of HA and β-TCP phases coexisting in BCP specimens. The residual was calculated by subtracting the calculated diffraction pattern from the experimental values.

The overall 3D structure of as-fabricated and sintered BCP scaffolds with different camphene contents was examined with optical microscopy. Their micro-scale porous structures were characterized using FE-SEM (JSM-6701F, JEOL Techniques, Tokyo, Japan) at various magnifications. The aligned macro-scale channels and the volume of the scaffolds were evaluated and calculated using micro-computed tomography (micro-CT, Skyscan 1173, Bruker, Billerica, MA, USA), further investigated with CT analysis software (Dragonfly software, Version 2024.1 for [Windows], Comet Technologies Canada Inc., Montréal, QC, Canada; software available at https://dragonfly.comet.tech/ (accessed on 30 August 2024)). A voxel size of 16.1 μm was used for image analyses. To analyze the overall porosity of scaffolds with different camphene contents, micro- and macroporosities were evaluated. The macroporosity (*P_Ma_*) was calculated using the CT analysis software, in which the volume of the macropore and the scaffold was taken into account, following the following equation:

(2) $$P_{Ma}\;\left[\%\right]=100-100\;\cdot \left(\frac{V_{s}}{V_{s}+V_{p}}\right)\;$$

*V_s_*: volume of the scaffold [mm^3^];

*V_p_*: volume of the macropore [mm^3^].

The microporosity (*P_Mi_*) of each scaffold with different camphene content was calculated using the volume computed with CT analysis software, their mass (*m*), and the true density (*ρ_t_*) of BCP ceramic (3.14 g/cm^3^, according to the manufacturer’s specification), following the following equation:

(3) $$P_{Mi}\;\left[\%\right]=100-100\;\cdot \left(\frac{m}{V_{s}+\rho_{t}}\right)$$

The overall porosity (*P_O_*) was then calculated with micro- and macroporosity, following the following equation:

(4) $$P_{O}\;\left[\%\right]=P_{Ma}+(100-P_{Ma})\;\cdot \left(\frac{P_{Mi}}{100}\right)$$

Five samples were examined by condition, and the results were calculated with consideration of the equations above to obtain the mean and standard deviation (*n* = 5).

### 2.7. Measurement of Compressive Strengths and Modulus

The mechanical properties of BCP scaffolds were evaluated using compressive strength tests, using samples with a diameter of ~7 mm and a height of ~4 mm. By using a universal testing machine (UTM; ST-1000, Salt Co., Ltd., Incheon, Republic of Korea), samples were compressed at a crosshead speed of 1 mm/min. The compressive load was applied parallel to the direction of macro-scale channels. The compressive strength and elastic modulus were calculated from the stress–strain curves. Measurements were performed on five samples to obtain the mean and standard deviation (*n* = 5).

### 2.8. Evaluation of Water Penetration Ability

The water penetration ability of the porous BCP scaffolds with varying camphene contents (40, 50, and 60 vol%) was evaluated by immersing their bottom surfaces in water containing red dye for enhanced visualization. Optical images of the scaffolds were captured at 10 and 30 s after immersion to assess the water penetration capabilities.

### 2.9. Statistical Analysis

Before performing all parametric statistics, the normality of distributions was verified with the Shapiro–Wilk test. All data were expressed as the mean ± standard deviation. Statistical analysis was performed using a one-way analysis of variance (ANOVA) with Tukey’s post hoc test using MATLAB (Release 2024b, The MathWorks, Inc., Natick, MA, USA). A *p*-value < 0.05 was considered statistically significant, which was graphically demonstrated with different superscripts within each figure.

## 3. Results and Discussion

### 3.1. Characterization of BCP Powders

**Figure 2:**
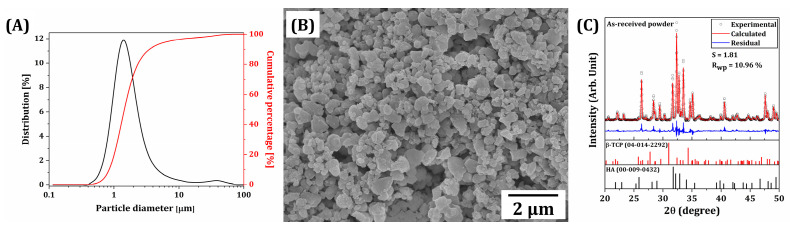
(**A**) Size distribution, (**B**) representative FE-SEM image, and (**C**) XRD pattern (refined by the Rietveld method) of BCP particles used for preparation of BCP suspensions for PS-DLP. In (**C**), the black circles indicate experimental values, the continuous red lines correspond to the calculated diffraction pattern, and the blue line represents the residual between the experimental and calculated values.

To manufacture dual-scale porosity BCP scaffolds with tightly controlled macroporous and microporous structures, commercially available BCP powders were used as received to prepare BCP suspensions for our PS-DLP. As-received BCP powders showed well-defined morphologies with submicron sizes (Figure 2A). Their particle size distribution was more closely examined by the laser diffraction method. The mean particle size (D_50_) was 1.46 ± 0.06 μm, while D_10_ and D_90_ were 0.84 ± 0.02 μm and 3.83 ± 0.92 μm, respectively. These submicron-sized powders would be effectively pushed by growing camphene-rich dendrites during the phase separation of BCP suspensions.

In addition, the crystalline phases of BCP powders were characterized by XRD, as shown in Figure 2C. A number of strong peaks corresponding to crystalline HA (JCPDS card no. 00-009-0432) and β-TCP (JCPDS card no. 04-014-2292) phases were observed. The contents of HA and β-TCP, calculated by Rietveld refinement, were 63.3 wt% and 36.7 wt%, respectively.

### 3.2. Phase Separation Behavior of BCP Suspensions

**Figure 3:**
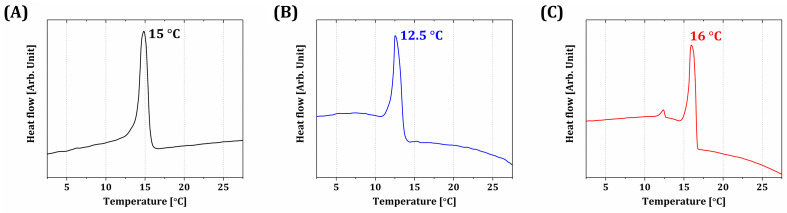
DSC curves obtained from BCP suspensions prepared using various camphene contents: (**A**) 40 vol%, (**B**) 50 vol%, and (**C**) 60 vol%.

To optimize the temperature for PS-DLP, the phase separation behaviors of BCP suspensions prepared using various camphene contents (40 vol%, 50 vol%, and 60 vol%) were characterized by DSC. All suspensions showed one strong exothermic peak, as shown in Figure 3, indicating the extensive crystallization of the camphene-rich phase [58,63]. In addition, similar peak temperatures (~15.0 °C, 12.5 °C, and 16.0 °C for the camphene contents of 40, 50, and 60 vol%, respectively) were observed. The peak temperature of the BCP suspension with 50 vol% camphene content showed the lowest among the three suspensions, which may be attributed by the balance between the content of camphene, TEGDMA, and PEGDA. However, it should be noted that the camphene crystal formation during the PS-DLP process (~5 °C) was uniform throughout the different contents of the camphene. Furthermore, it should also be stated that achieving similar peak temperatures of suspensions with different camphene contents is one of the most striking advantages of TEGDMA/PEGDA blends compared to the TEGDMA monomer alone, since similar crystallization behavior of camphene could be achieved throughout BCP suspensions with different camphene contents [63]. Also, it should be stated that condensation of water vapor during the PS-DLP process could happen if the phase separation point is too low, since the platform should remain at a lower temperature than the phase separation points to induce camphene crystal formation. A previous study has shown that when TEGDMA is employed to dissolve solid camphene, the phase separation point of camphene–TEGDMA solutions decreases notably with a decrease in camphene content, thus making it troublesome to obtain the low porosities required for high mechanical properties [63]. For that reason, PEGDA with lower solubility of camphene compared to TEGDMA was employed as an anti-solvent. Since PEGDA can be homogeneously mixed with TEGDMA, a uniform camphene–TEGDMA/PEGDA mixture could be achieved. Therefore, the use of TEGDMA/PEGDA blends, as well as control over their compositional ratio, allows the phase separation of all BCP suspensions prepared using various camphene contents at moderate temperatures (e.g., 5 °C used in this study) that can be readily achieved using conventional thermoelectric modules, thus providing favorable conditions for our PS-DLP process.

### 3.3. Rheological Behaviors of BCP Suspensions

**Figure 4:**
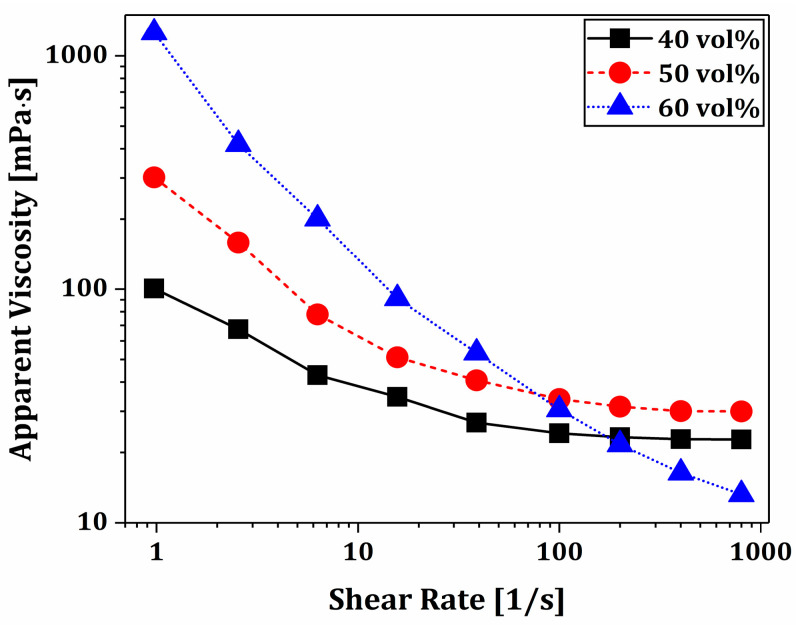
Apparent viscosities of BCP suspensions prepared using various camphene contents (40, 50, and 60 vol%) as a function of shear rate.

To make full use of PS-DLP for manufacturing dual-scale porosity BCP scaffolds with a microporous hexagonal structure, BCP suspensions should have desired rheological behaviors. To this end, we employed “DISPERBYK-180”, an alkylolammonium salt of a copolymer with acidic groups as the dispersant, and the optimized content (5 wt% with respect to the BCP content) was utilized based on the previous study to achieve a homogeneous suspension after mixing with a planetary centrifugal mixer [63]. The apparent viscosities of BCP suspensions were plotted as a function of shear rate, as shown in Figure 4. All suspensions exhibited that the apparent viscosity decreased exponentially with an increase in shear rate, which is a typical characteristic of the shear-thinning behavior of highly concentrated ceramic suspensions. They showed reasonably low viscosities (<35 mPa·s) at high shear rates (>100/s). This finding suggests that BCP powders can be uniformly mixed with TEGDMA/PEGDA–camphene solutions using a conventional planetary centrifugal mixer, and BCP suspensions prepared in this way can be uniformly spread onto build platforms by a recoating system for the PS-DLP process. In addition, sufficiently high viscosities at a low shear rate of 1/s were observed −100.6 mPa·s, 301.8 mPa·s, and 1257.5 mPa·s for the camphene contents of 40 vol%, 50 vol%, and 60 vol%, respectively. Thus, it is reasonable to suppose that the thin layers of BCP suspension can be maintained well and then turn into a gel-like state after phase separation at 5 °C for the PS-DLP process, since camphene-rich crystals can make solid networks surrounded by networks composed of liquid TEGDMA/PEGDA monomers enclosing BCP powders. However, it should be noted that higher camphene content results in higher apparent viscosities at relatively low shear rates (<100/s), which should be more deeply interpreted in further studies to establish protocols for the formulation of phase-separable, photocurable ceramic suspensions for PS-DLP.

### 3.4. Photopolymerization Behaviors of Phase-Separated BCP Layers

**Figure 5:**
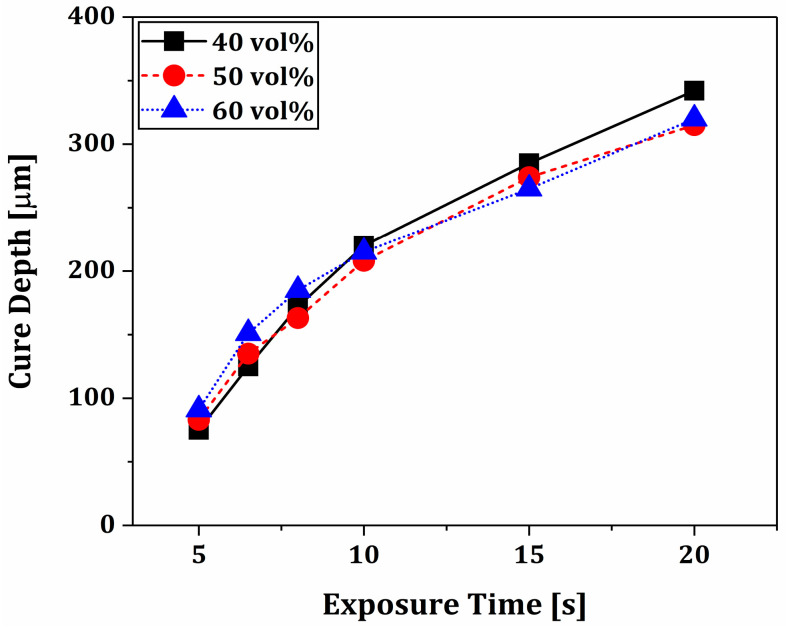
Photocured thicknesses of phase-separated BCP layers produced using various camphene contents (40, 50, and 60 vol%) as a function of UV illumination time. The phase separations of BCP suspensions were carried out at 5 °C prior to photocuring.

Unlike the conventional DLP process using flowable ceramic suspensions, our PS-DLP should photopolymerize gel-like, phase-separated BCP layers due to a three-dimensional network of camphene-rich crystals. Thus, the photopolymerization behaviors of phase-separated BCP layers should be carefully characterized to optimize the UV illumination time for PS-DLP. To this end, the thin layers of BCP suspensions prepared using various camphene contents (40 vol%, 50 vol%, and 60 vol%) underwent phase separation at 5 °C using our system and were then photocured for various UV illumination times (5–20 s). After which, their photocured thicknesses were measured by a micrometer, and the results are displayed in Figure 5. They showed similar trends, where the photocured thickness (often termed as “cure depth”) increased with an increase in UV illumination time, as expected. This finding suggests that the presence of camphene-rich crystals would not seriously deteriorate the photopolymerization behaviors of TEGDMA-PEGDA monomers enclosing BCP powders. In addition, defect-free bonding between phase-separated BCP layers can be obtained by optimizing the UV illumination time for the DLP process when considering the layer thickness determined by the recoating and phase separation processes. Based on this finding, we employed a UV illumination time of 6.5 s to completely photocure 100 μm-thick phase-separated layers for manufacturing dual-scale porosity BCP scaffolds.

### 3.5. Macrostructures and Microstructures of As-Manufactured Macroporous BCP Scaffolds

**Figure 6:**
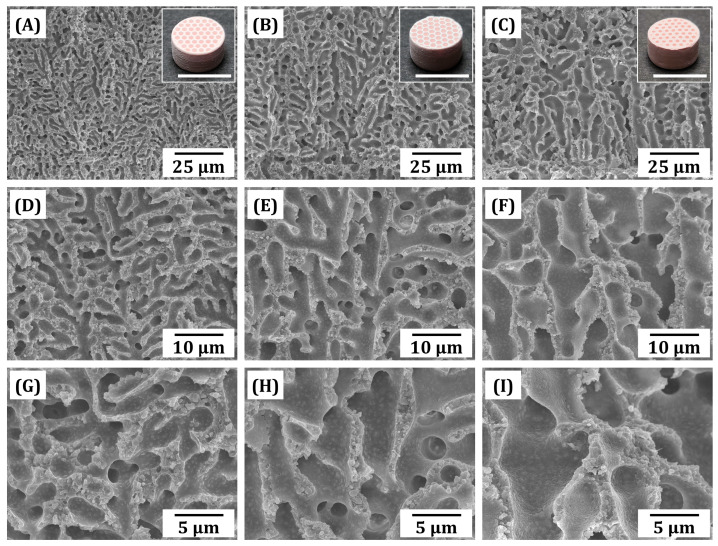
FE-SEM images of fracture surfaces of green dual-scale porosity BCP scaffolds manufactured using various camphene contents: 40 vol% (**A**,**D**,**G**), 50 vol% (**B**,**E**,**H**), and 60 vol% (**C**,**F**,**I**). Insets in (**A**–**C**) show optical images of as-manufactured BCP scaffolds (scale = 10 mm).

The macrostructures and microstructures of as-manufactured macroporous BCP scaffolds were closely examined by OM and FE-SEM, as shown in Figure 6. All scaffolds showed tightly controlled honeycomb structures without noticeable defects, such as delamination between printed layers (insets in Figure 6A–C). The pinkish color of as-manufactured scaffolds represents the presence of the inert dye used to enhance the printing resolution. It should be noted that the height of the samples was as high as ~ 6 mm, suggesting that our PS-DLP can be used to manufacture large bone scaffolds for repairing large bone defects without difficulty. During the PS-DLP process, BCP suspensions with different camphene contents remained homogeneous after mixing in a planetary centrifugal mixer, due to the appropriate amount of dispersant employed to formulate BCP suspensions. In addition, all as-manufactured scaffolds showed that pores were uniformly formed within BCP frameworks and at interfaces between printed layers (Figure 6A–I). In addition, the pores were highly elongated from the bottom to the top of the scaffolds (Figure 6D–F). This finding suggests that camphene-rich crystals can grow preferentially along the heat conduction direction due to the cool build platform placed at 5 °C. However, the fraction and the pore size increased with an increase in camphene content, as is often the case with the camphene-based freeze casting used for manufacturing porous ceramics [28,29,59,61,63]. Regardless of the camphene content, all photopolymerized frameworks revealed relatively dense microstructure without defects (e.g., large voids and cracks), where BCP particles were uniformly embedded within the photopolymerized phase (Figure 6G–I). These findings suggest that our PS-DLP technique enables the construction of dual-scale porosity BCP scaffolds with tailored microporous frameworks.

### 3.6. Macrostructures of Sintered Dual-Scale Porosity BCP Scaffolds

**Figure 7:**
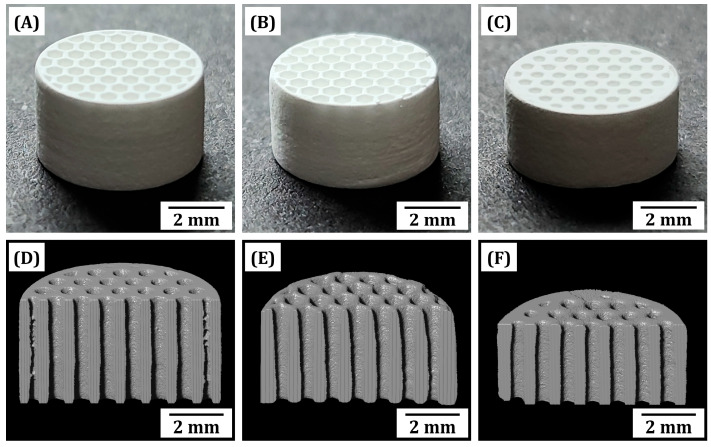
Optical images (**A**–**C**) and μ-CT images (**D**–**F**) of sintered dual-scale porosity BCP scaffolds produced using various camphene contents: 40 vol% (**A**,**D**), 50 vol% (**B**,**E**), and 60 vol% (**C**,**F**). Sintering was conducted at 1150 °C for 3 h.

As-manufactured macroporous BCP scaffolds were heat-treated for debinding according to a carefully designed heat-treatment schedule (e.g., heating rate, target temperature, and dwelling time) and then sintered at 1150 °C for 3 h for the densification of BCP particles comprising microporous frameworks. All BCP scaffolds showed well-defined honeycomb structures without any noticeable defects, such as cracks at interfaces between printed layers and within printed layers, and severe distortion (Figure 7A–C). This finding suggests that a large amount of the photopolymerized phase in BCP frameworks can be completely removed without deteriorating the highly packed BCP particles, and thus BCP particles can be highly densified. Their internal pore structures examined by μ-CT revealed that straight channels separated by BCP frameworks were well constructed (Figure 7D–F). Note that once again, the homogeneity of BCP suspensions during the PS-DLP process was shown, since precipitation of BCP particles during the PS-DLP process would induce heterogeneous solid content in the BCP frameworks between the bottom and top of the specimen. The difference in solid content would distort the framework during heat-treatment; however, it should be emphasized that both the framework and overall scaffold remained straight, as shown in Figure 7.

Linear sintering shrinkages of dual-scale porosity BCP scaffolds measured in the x- and z-directions are summarized in Table 3. As the camphene content increased from 40 vol% to 60 vol%, the linear shrinkage increased from 21.9 ± 0.4% to 26.2 ± 0.5% in the x-direction and 23.0 ± 0.4% to 28.7 ± 0.6% in the z-direction. It is reasonable to suppose that higher sintering shrinkage is attributed to higher shrinkage of pores surrounding densified walls, as is often the case in the freeze casting of porous ceramics [67,68]. On the other hand, slightly larger sintering shrinkages in the z-direction would be attributed to the sublimation and/or evaporation of camphene used in this study. This issue would be interpreted in further studies.

### 3.7. Microporous Structure of Sintered BCP Frameworks

**Figure 8:**
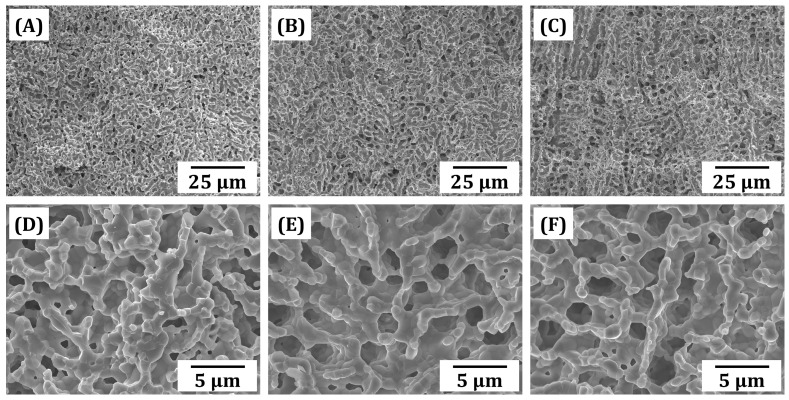
Representative FE-SEM images of fracture surfaces of sintered BCP frameworks obtained using various camphene contents: 40 vol% (**A**,**D**), 50 vol% (**B**,**E**), and 60 vol% (**C**,**F**).

The microporous structures of BCP frameworks produced using various camphene contents (40 vol%, 50 vol%, and 60 vol%) after sintering at 1150 °C for 3 h were examined by FE-SEM, and their representative FE-SEM images are displayed in Figure 8A–F. Regardless of the camphene content, a number of open, interconnected pores were formed uniformly throughout the BCP frameworks (Figure 8A–C). However, the pore size decreased remarkably after sintering, since pores shrunk extensively due to the densification of BCP particles comprising BCP frameworks. As expected, higher microporosity was obtained using higher camphene contents. It is noteworthy that BCP walls comprising BCP frameworks could be almost fully densified (Figure 8D–F), which is one of the most striking advantageous features of our PS-DLP technique for the manufacturing of porous ceramics. In other words, during phase separation, BCP particles dispersed in liquid photocurable monomers are pushed by growing camphene-rich crystals and then concentrated. Thus, they can be highly densified after sintering at proper temperatures. The pore interconnectivity increased with an increase in camphene content due to an increase in porosity. This finding suggests that micron-sized pores would provide favorable paths for the transport of body fluids including blood, growth factors, oxygen, and nutrients for new bone tissue formation, while dense BCP walls effectively withstand applied loads during bone regeneration [47,69,70].

### 3.8. Crystalline Phases of Sintered BCP Scaffolds

**Figure 9:**
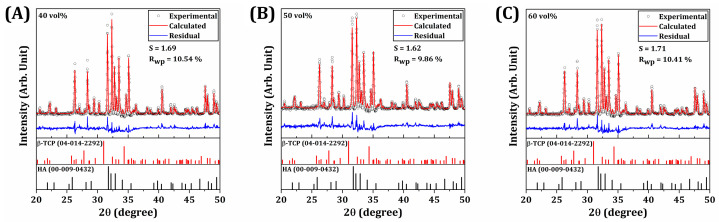
XRD patterns of sintered BCP scaffolds with various camphene contents (**A**) 40 vol%, (**B**) 50 vol%, and (**C**) 60 vol% refined by the Rietveld method. The black circles indicate experimental values, the continuous red lines correspond to the calculated diffraction pattern, and blue line represents the residual between the experimental and calculated values.

XRD analysis of the sintered scaffolds (Figure 9) revealed the presence of HA and β-TCP phases within. The increased formation of β-TCP phase is attributed to the phase transformation of HA during sintering at 1150 °C [71,72], when comparing to the BCP powder employed in this work (Figure 2C). Rietveld refinement results (Table 4) showed that the HA content of BCP scaffolds decreased from 63.3 wt% to ~48–49 wt% when comparing to the as-received powder, with a corresponding increase in β-TCP content, which is due to decomposition of the HA phase to β-TCP phase at the relatively high temperature of 1150 °C [3,4,5]. Note that the sintering temperature could be altered to control the content of β-TCP phase to induce or to prevent further degradation in vivo. However, the sintering condition should be carefully controlled since it can significantly affect pore morphology, which has a high impact on both microporosity and mechanical strength. Furthermore, the content of HA and β-TCP contents of BCP scaffolds with different camphene contents (40, 50, and 60 vol%) were similar, in a close range, since the scaffolds were sintered at the same temperature at 1150 °C for 3 h.

### 3.9. Overall Porosities, Macroporosities, and Microporosities of Dual-Scale Porosity BCP Scaffolds

**Figure 10:**
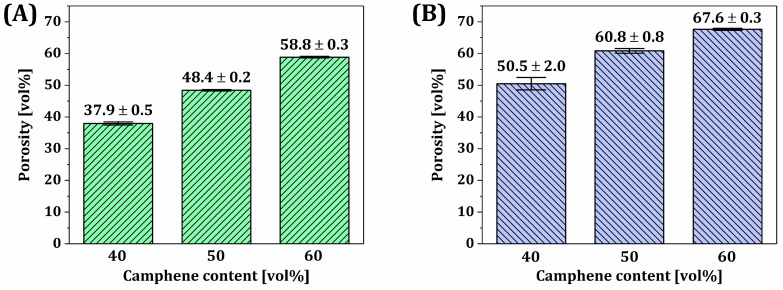
(**A**) Microporosities and (**B**) overall porosities of dual-scale porosity BCP scaffolds manufactured using various camphene contents (40 vol%, 50 vol%, and 60 vol%). Green bars in (**A**) represent microporosity, while blue bars in (**B**) represent overall porosity of the corresponding samples.

The bone regeneration ability and mechanical properties of dual-scale porosity BCP scaffolds should be strongly influenced by their porous structures. Thus, their overall porosities, macroporosities (i.e., fraction of unidirectional pores and channels), and microporosities (i.e., fraction of micron-sized pores in the BCP frameworks) were computed by μ-CT analyses using a high resolution with a voxel size of 16.1 μm. Regardless of the camphene content, all BCP scaffolds showed similar macroporosities without significant discrepancies—20.2 ± 2.6 vol%, 24.1 ± 1.7 vol%, and 21.3 ± 1.0 vol% for the camphene contents of 40 vol%, 50 vol%, and 60 vol%, respectively. On the other hand, the microporosities increased remarkably, showing linear increasing trend from 37.9 ± 0.5 vol% to 58.8 ± 0.3 vol% with an increase in camphene content from 40 vol% to 60 vol% (Figure 10A). This finding suggests that our PS-DLP can precisely tailor the microporosity of BCP frameworks as suggested in our previous works [63]. However, the measured values were slightly lower than the initial camphene contents, since camphene-rich crystals would contain a certain amount of liquid photocurable monomers (i.e., TEGDMA and PEGDA) based on their phase diagrams—high and low dissolution behaviors in TEGDMA and PEGDA, respectively [63]. On the other hand, dual-scale porosity BCP scaffolds manufactured with higher camphene contents had higher overall porosities—50.5 ± 2.0 vol%, 60.8 ± 0.8 vol%, and 67.6 ± 0.3 vol% for the camphene contents of 40 vol%, 50 vol%, and 60 vol%, respectively. It should be noted that this increase is attributed to an increase in microporosity.

### 3.10. Mechanical Properties of BCP Scaffolds

**Figure 11:**
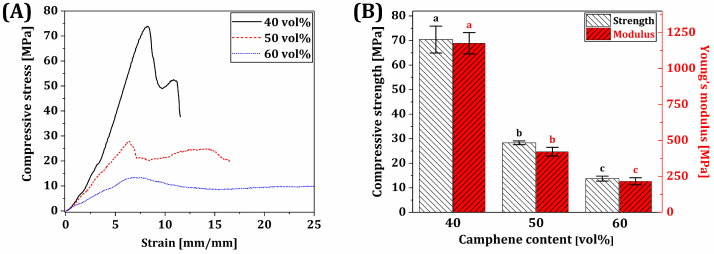
(**A**) Representative compressive stress–strain curves and (**B**) compressive strengths and modulus of dual-scale porosity BCP scaffolds manufactured using various camphene contents (40, 50, and 60 vol%). Different letters in each color represent statistical significance (*p*-value < 0.05).

To evaluate the potential of dual-scale porosity BCP scaffolds with a honeycomb structure for replacing bones, their mechanical properties were characterized by compressive strength tests. Figure 11A shows the representative compressive stress–strain curves of dual-scale porosity BCP scaffolds manufactured using various camphene contents (40 vol%, 50 vol%, and 60 vol%). All scaffolds showed that the stress increased almost linearly due to their elastic deformation and then decreased due to the brittle fracture of BCP frameworks, as is often the case with porous ceramic scaffolds [73,74,75]. However, they showed different stress–strain responses after reaching the maximum points attributed to different fracture strengths of BCP frameworks. More specifically, in the case of the lowest camphene content of 40 vol%, the compressive stress decreased rapidly and then slightly increased with an increase in strain. This fracture behavior is attributed to the fact that several strong BCP frameworks would be initially fractured by the maximum stress applied [43,75,76,77,78]; however, their neighboring frameworks survived and retained additional compressive stress to a certain extent, preventing the overall structural failure. On the other hand, when relatively high camphene contents (50 vol% and 60 vol%) were employed, dual-scale porosity BCP scaffolds showed small changes in compressive stresses even for considerable strains. It is reasonable to suppose that BCP frameworks with relatively low fracture strengths would be crushed under compression, as is often the case of highly porous ceramics [77,78,79]. The compressive strengths and modulus of dual-scale porosity BCP scaffolds are displayed in Figure 11B. As the camphene content increased from 40 vol% to 60 vol%, the compressive strengths and modulus decreased from 70.4 ± 5.5 MPa to 13.7 ± 1.0 MPa and 1175.9 ± 74.0 MPa to 215.9 ± 24.7 MPa, respectively. These reductions are mainly attributed to a decrease in the fracture strength of BCP frameworks caused by an increase in microporosity. However, it should be noted that the compressive strength of dual-scale porosity BCP scaffolds can be tailored simply by adjusting the camphene content used for our PS-DLP. More specifically, a range of compressive strengths (~14–70 MPa) obtainable via our PS-DLP would find useful uses to replace not only load-bearing bones but also non-load-bearing bones, while providing macro/microporous structures with high porosities. This finding suggests that our PS-DLP technique could allow for the manufacturing of a variety of dual-scale porosity ceramic structures with high compressive strengths and porosities, which could be utilized in various applications such as bone scaffolds, structural components, and electronical parts [80,81,82].

### 3.11. Mass Transport Abilities of Dual-Scale Porosity BCP Scaffolds

**Figure 12:**
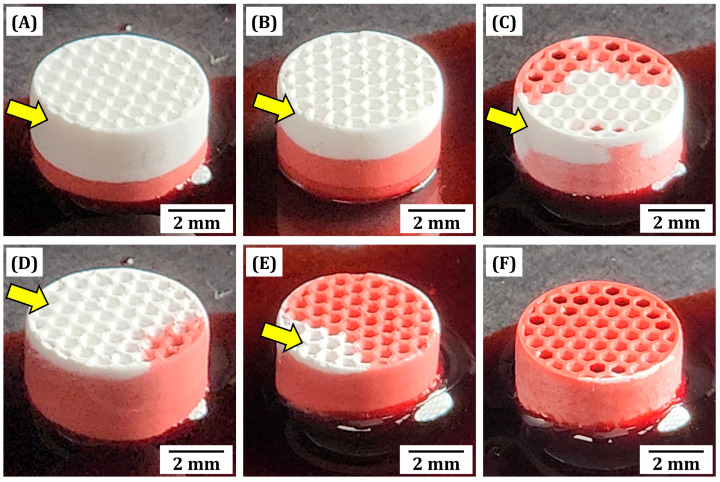
Optical images of sintered BCP scaffolds manufactured using various camphene contents after different immersion times: 40 vol% (10 s (**A**), 30 s (**D**)), 50 vol% (10 s (**B**), 30 s (**E**)), and 60 vol% (10 s (**C**), 30 s (**F**)). Red dye was added to the water for enhanced visualization. Yellow arrows indicate regions without water penetration.

To evaluate the utility of dual-scale porosity BCP scaffolds, particularly those using microporous frameworks for transporting bone fluids, blood, growth factors, oxygen, and nutrients, qualitative characterization was roughly performed. The bottoms of BCP scaffolds were immersed in water for 10 s and 30 s and the changes in their optical appearances were directly monitored by optical microscopy. For visualization, a small amount of red dye was added into water. After immersion for 10 s, all scaffolds showed that their lower parts were filled with water since water can penetrate through both microchannels and micropores in BCP frameworks (Figure 12A–C). However, the degree of water penetration increased remarkably with an increase in camphene content, similar to the previous study which employed a gyroid structure as a microporous framework [29]. The increased water penetration ability with similar framework geometry indicates the importance of tuned microporosity within BCP frameworks. Particularly, the upper part of BCP scaffolds produced using the highest camphene content (60 vol%) was partially infiltrated by water, suggesting much higher mass transport ability due to greater microporosity (Figure 12C). This trend became more obvious after immersion for 30 s (Figure 12D–F). All regions of BCP scaffolds produced using the highest camphene content (60 vol%) were infiltrated by water (Figure 12F). This finding suggests that the creation of micropores in BCP frameworks can provide favorable paths for the transport of masses, including body fluids, blood, growth factors, oxygen, and nutrients, thus promoting faster bone tissue regeneration when used as bone scaffolds [83]. In addition, the mass transport ability of dual-scale porosity BCP scaffolds can be significantly enhanced by increasing the microporosity of BCP frameworks using our PS-DLP. Although the mass transport ability of dual-scale porosity BCP scaffolds were roughly evaluated, further biological assessments regarding the in vivo bone tissue regeneration performance of BCP scaffolds such as biodegradation and osseointegration remains to be explored.

## 4. Conclusions

Dual-scale porosity BCP scaffolds with a honeycomb structure composed of microporous walls were manufactured using our newly developed PS–DLP process. Particularly, the microporosity of BCP walls could be readily tailored by adjusting the camphene content employed in BCP suspensions. Consequently, a range of mechanical properties (~13.7–70.4 MPa) with varying microporosities (~59–38 vol%) could be obtained. In addition, interconnected micropores in BCP walls notably facilitated the transport of water. Thus, the dual-scale porosity BCP scaffolds proposed in this study would be used to replace not only non-load-bearing but also load-bearing bones.

## Figures and Tables

**Table 1:** Weight fractions of camphene, TEGDMA, and PEGDA in camphene–TEGDMA/PEGDA blend in this work to prepare BCP suspensions with various camphene contents (40, 50, and 60 vol%) for phase separation-assisted DLP 3D printing.

Camphene Content * [vol%]	Camphene [wt%]	TEGDMA [wt%]	PEGDA [wt%]
40	46.7	35.7	17.6
50	57.0	36.9	6.1
60	66.7	33.3	0

*: For all BCP suspensions, the solid loading excluding camphene was 33.8 vol%.

**Table 2:** Schedule for debinding and sintering.

Heat-Treatment	Debinding							Sintering
Step	1	2	3	4	5	6	7	8
Heating rate [°C/min]	1	1	1	1	1	1	1	5
Temperature [°C]	140	205	220	250	270	330	440	1150
Dwelling time [min]	60	60	60	60	60	60	60	180

**Table 3:** Linear sintering shrinkages of BCP scaffolds with various camphene contents (40, 50, and 60 vol%) in the x- and z-directions.

Camphene Content [vol%]	40	50	60
Shrinkage [%] (in x-direction)	21.9 ± 0.4	23.8 ± 0.5	26.2 ± 0.5
Shrinkage [%] (in z-direction)	23.0 ± 0.3	27.5 ± 0.5	28.7 ± 0.6

**Table 4:** Weight fraction results of crystalline phases for BCP scaffolds with various camphene contents (40, 50, and 60 vol%) calculated with Rietveld refinement.

	HA Content [wt%]	β-TCP Content [wt%]
As-received powder	63.3	36.7
40 vol%	49.0	51.0
50 vol%	48.6	51.4
60 vol%	47.6	52.4

## Data Availability

The original contributions presented in this study are included in the article. Further inquiries can be directed to the corresponding author.
